# Changes in Nigeria’s enabling environment for nutrition from 2008 to 2019 and challenges for reducing malnutrition

**DOI:** 10.1007/s12571-022-01328-2

**Published:** 2022-11-22

**Authors:** Olutayo Adeyemi, Mara van den Bold, Nicholas Nisbett, Namukolo Covic

**Affiliations:** 1 Department of Human Nutrition and Dietetics, Faculty of Public Health, University of Ibadan, Ibadan, Nigeria; 2 Formerly of the International Food Policy Research Institute, Washington D.C., USA; 3 Clark University, Worcester, MA USA; 4 Institute of Development Studies, Brighton, UK; 5 International Livestock Research Institute, Addis Ababa, Ethiopia

**Keywords:** Nutrition, Capacity, Political commitment, Coordination, Policy analysis

## Abstract

Key 2025 global nutrition targets are unlikely to be met at current rates of progress. Although actions necessary to reduce undernutrition are already mostly known, knowledge gaps remain about how to implement these actions in contextually appropriate ways, and at scales commensurate with the magnitude of the problem. This study describes the nutrition enabling environment in Nigeria, a country that contributes significantly to the global undernutrition burden, and identifies potential entry points for improving the enabling environment that could facilitate implementation and scale-up of essential intervention coverage. Study data were obtained from two sources: content analysis of 48 policies/strategies from agriculture, economic, education, environment, health, nutrition, and water/sanitation/hygiene sectors; and interviews at federal level (16) and in two states (Jigawa (10) and Kaduna (9) States). The study finds that aspects of the enabling environment improved between 2008 and 2019 and facilitated improvements in implementation of nutrition-specific and nutrition-sensitive interventions. Enabling environment components that improved included the framing of nutrition as a multisectoral issue, nutrition advocacy, political attention, evidence around intervention coverage, civil society involvement, and activity of nutrition champions. These factors have been especially important in creating and sustaining momentum for addressing malnutrition. While challenges remain in these aspects, greater challenges persist for factors needed to convert momentum into improvements in nutrition outcomes. Research and data that facilitate shared understanding of nutrition; improved multisectoral and vertical coordination; increased and improved delivery and operational capacity; and increased resource mobilization will be especially important for achieving future progress in nutrition in Nigeria.

**Supplementary Information:**

The online version contains supplementary material available at 10.1007/s12571-022-01328-2.

## Introduction

Globally, undernutrition has declined over the past few decades. However, a considerable undernutrition burden remains to be addressed and progress in many countries has been slow (Development Initiatives, 2017; Onis et al., 2000). To accelerate needed progress, the World Health Assembly (WHA) ratified six nutrition goals in 2012 with a timeline of 2025 (McGuire, 2015). The establishment of these goals, amongst other factors (e.g., the launch of the Scaling Up Nutrition (SUN) movement) and explicit inclusion of nutrition in the Sustainable Development Goals (SDGs)), triggered increased global attention to addressing high rates of maternal and child undernutrition. Yet more than halfway to the timeline set by the WHA, progress remains limited (Bhutta et al., 2008, 2013; Bryce et al., 2008; Heidkamp et al., 2021; Ruel et al., 2013; Victora et al., 2021).

Answers to questions about *whether* to enact and implement nutrition policies (agenda setting) or *what* to implement (actions necessary to minimize the prevalence of undernutrition – nutrition-specific and nutrition-sensitive interventions) are well-known. Acting for nutrition (*how* to achieve policy implementation) remains the major challenge (Gillespie et al., 2015), including in Nigeria (MBNP, 2016). Indeed, three factors are imperative for reducing malnutrition in any population – 1) interventions with meaningful effect sizes on outcomes; 2) widespread coverage of these interventions; and 3) sustainability of high intervention coverage (Hawe et al., 1997). Achieving and sustaining high coverage of effective multisectoral interventions remain the critical limiting factors for reducing malnutrition (Gillespie et al., 2013; Heidkamp et al., 2021).

An enabling environment for nutrition has been characterised as political and policy processes that create and sustain a drive to effectively and continuously implement actions that reduce undernutrition (Gillespie et al., 2013). Several authors (Bhutta et al., 2020; Gillespie et al., 2017; Headey et al., 2017) have studied nutrition enabling environments and undernutrition actions in countries considered to be success stories or exemplars in reducing stunting. These studies and associated reviews (Heidkamp et al., 2021) highlight that leadership; availability of relevant, quality data at lower administrative levels; multisectoral and vertical coherence; operational capacity; and finance are key enabling environment factors for successful nutrition intervention delivery. Other authors (Baker et al., 2018, 2019; Fracassi et al., 2020) emphasize that there are five forms of political commitment that are indispensable for nutrition – rhetorical, institutional, operational, embedded, and system-wide commitment. These forms of commitment interact dynamically and improvements or declines in one form of commitment influences improvements or declines in other forms, respectively. The authors (Baker et al., 2018, 2019; Fracassi et al., 2020) further indicate that nutrition enabling environments are constrained when some forms of commitment exist while other forms are limited. An understanding of nutrition enabling environments is thus required to characterise contextual forms of political commitment and identify entry points for strengthening such commitment (Baker et al., 2018, 2019; Fracassi et al., 2020).

Given the slow global progress towards achieving the WHA nutrition goals (Victora et al., 2021), as well as the negative effects of the COVID-19 pandemic on past and present nutrition gains (Headey et al., 2020; Osendarp et al., 2021), it is important to better understand how nutrition enabling environments can be improved to accelerate nutrition actions.

Progress towards reducing undernutrition has been particularly limited in West Africa, including in Nigeria, which bears a sizeable proportion of global malnutrition (UNICEF et al., 2021). Recent improvements in undernutrition determinants in Nigeria have not been sufficient to produce considerable reductions in undernutrition (Adeyemi et al., 2022). Overall, the prevalence of wasting halved between 2008 and 2018, stunting declined by 9.4%, and maternal undernutrition did not improve (Table 1). Progress in nutrition determinants was variable with some improving (e.g., exclusive breastfeeding, vaccinations, antenatal care, and other health services), while others deteriorated (e.g., food security and child illnesses, Table 1). Even for determinants that improved, the levels in 2018/2019 were inadequate. For instance, in 2018, less than 50% of births occurred in a health facility and less than 25% of children had received all age-appropriate vaccinations, even though child vaccinations had increased by more than 100% between 2008 and 2018 (Adeyemi et al., 2022). These findings suggest that interventions to address nutrition have been insufficient, indicating a limited nutrition enabling environment in Nigeria.

**Table 1:** Selected Nutrition Outcomes and Determinants in Nigeria, 2008/2009 and 2018/2019^a^

**Indicators**	**2008/2009**	**2018/2019**
**Outcomes**		
Prevalence of wasting, weight for height < -2 SD (% of children under 5)^b^	13.9	6.8
Prevalence of stunting, height for age < -2 SD (% of children under 5)^b^	40.6	36.8
Prevalence of overweight, weight for height > 2 SD (% of children under 5)^b^	8.8	2.1
Prevalence of underweight, body mass index < 18.5 kg/m^2^ (% of women ages 15–49)	12.2	12.1
Prevalence of short stature, height < 145 cm (% of women ages 15–49)	3.0	1.4
Prevalence of anaemia among children (% of children under 5)	73.6	68.9
Prevalence of anaemia among women of reproductive age (% of women ages 15–49)	55.9	55.1
**Immediate Determinants**		
Exclusive breastfeeding (% of children under 6 months)	12.5	28.7
Completed all basic vaccinations by 12 months (% of children ages 12–23 months)^b^	19.2	28.3
Vitamin A supplementation coverage rate (% of children ages 6–59 months)	78.0	80.0
Incidence of malaria (per 1,000 population at risk)	424.7	291.9
Prevalence of fever among children (% of children under 5)^b^	15.9	24.2
Prevalence of diarrhoea among children (% of children under 5)^b^	10.1	12.8
**Underlying Determinants**		
Prevalence of undernourishment (% of population)	6.8	14.6
Use of insecticide-treated bed nets (% of under-5 population)	5.5	52.2
Pregnant women receiving skilled prenatal care (%)^b^	57.7	67.0
Births attended by skilled health staff (% of total)	34.4	43.3
People using safely managed drinking water services (% of population)	17.1	21.3
People using safely managed sanitation services (% of population)	24.1	29.9
**Basic Determinants**		
Fertility rate, total (births per woman)	5.9	5.3
Literacy rate, adult female (% of females ages 15 and above)	41.4	52.7
Poverty headcount ratio at $3.20 a day (2011 PPP) (% of population)	79.5	71.0
Gini index (World Bank estimate)	43.0	35.1
Age dependency ratio (% of working-age population)	87.7	86.7
Urban population (% of total population)	41.7	51.2
Women participating in the three decisions (own health care, major household purchases, and visiting family, % of women age 15–49)	32.1	33.5

^a^Unless otherwise indicated, all data retrieved from the World Development Indicators updated 23 November 2021

^b^Retrieved from NPC and ICF (2019) and NPC and Macro (2009)

The Stories of Change study (SoC) in Nigeria, part of Transform Nutrition West Africa project (TNWA 2017–2021)^1^, focused on identifying how to better support policy and programme decisions and actions to accelerate reductions in maternal and child undernutrition in the country.^2^ The Nigeria SoC assessed the enabling environment to understand how nutrition improves or does not improve in a context which, unlike previous studies of nutrition enabling environments, is yet to be a nutrition success story. We also aim to contribute to the collective understanding of nutrition enabling environments. Specifically, the objectives of our study were to:

1. Describe changes in the enabling environment for nutrition over time that may have contributed to changes in malnutrition in Nigeria.
2. Identify possible areas for improvement in the enabling environment that could facilitate increased political commitment and scaling up of essential intervention coverage.

Deriving from existing literature (Baker et al., 2018, 2019; Fracassi et al., 2020; Gillespie et al., 2013), our study assumes that nutrition political commitment and enabling environment are constantly interacting and influencing each other in context-specific ways; and that addressing the enabling environment for nutrition is key for political commitment building and effective actions to reduce malnutrition.

## Enabling environment framework

Our study adopts the nutrition enabling environment framework by Gillespie et al. (2013), which conceptualizes three categories of factors – narratives, knowledge, and evidence; political economy and governance; and capacity and resources – that are necessary features of enabling environments. These factors have been identified as fundamental for effectively implementing nutrition interventions and improving intervention quality and coverage (Gillespie et al., 2013; Gillespie & van den Bold, 2015). For each category of factors, the framework further specifies factors necessary for creating and sustaining momentum for reducing malnutrition, and factors needed for converting momentum into results (Gillespie et al., 2013).

### Enabling narratives, knowledge, and evidence

For creating and sustaining momentum, narratives are enabling when there is reliable and timely data about nutrition determinants at programmatic level, actors working on nutrition align around common narratives and framing of nutrition issues, and these actors advocate and communicate with external audiences (including decision makers) using language that effectively resonates with the audiences (Baker et al., 2018; Gillespie et al., 2013; Shiffman & Smith, 2007). Enabling framing may manifest as nutrition policy integration, in which nutrition is holistically addressed across the policy goals and instruments of all relevant government ministries, departments, and agencies, spanning multiple sectors (Namugumya et al., 2020b). Enabling the conversion of momentum to nutrition results involves implementation research to understand the mechanisms through which interventions are contextually effective, and the use of evidence-based approaches in intervention scale-up (Gillespie et al., 2013).

### Enabling political economy and governance

Nutrition political economy is enabling for creating and sustaining momentum when nutrition actors (individuals and organizations) have clearly defined and understood roles and there is effective coordination among multisector actors (Gillespie et al., 2013). Role ambiguity can result in policies/strategies becoming low priority among actors and remaining largely unimplemented (Sabatier & Mazmanian, 1980; Sawicki et al., 2019). Effective multisectoral coordination occurs when persons vulnerable to malnutrition concurrently receive interventions that address the multifaceted causes of malnutrition (Benson, 2007; Haddad et al., 2004). There are several mechanisms, requiring varying degrees of convergence, that can be used to achieve coordinated intervention delivery (Harris & Drimie, 2012; Kim et al., 2017). Even coordination involving lower degrees of convergence can be enabling for nutrition through co-location of interventions (Heidkamp et al., 2021; Levinson et al., 2013). Nutrition political economy and governance is also enabling for creating momentum when there is political attention for nutrition and actors are effectively held accountable for action/inaction and performance (Gillespie et al., 2013). For conversion of momentum to results, political economy is enabling when subnational governments adapt national policies to their own contexts and are committed to delivering interventions to beneficiaries with high fidelity to intervention design. Civil society engagement for conducting advocacy, facilitating accountability, generating contextual knowledge, and delivering services is further enabling, as is the harnessing of the private sector to achieve optimal nutrition outcomes through goods and services demanded of and supplied by the private sector (Baker et al., 2018; Gillespie et al., 2013).

### Enabling capacity and resources

Strong leaders and champions, backed by systemic and organizational capacity, are required to create and sustain momentum. Also needed are estimates of the costs and economic benefits of addressing specified nutrition challenges. For converting momentum to results, an enabling environment will sequence and prioritize needed nutrition actions, especially in contexts where it is not feasible to address all nutrition determinants concurrently. Different types of capacity, including strategic, structural, role, financial, operational (infrastructure and staff mix), and delivery (methods and skills) capacity, available at all levels of planning and implementation, is imperative for enabling environments (Bryce et al., 2008; Gillespie et al., 2013; Pelletier et al., 2011; Potter & Brough, 2004). Relatedly, nutrition training programs and academic curricula must be of high quality and deployed to increase and sustain nutrition human resources (Baker et al., 2018; Gillespie et al., 2013). Some authors have emphasized that training programmes can only be effective when they are used as part of a costed national nutrition workforce strategy and implementation plan that clearly defines required competencies that are linked to job performance, qualification processes, roles and responsibilities for delivering trainings, and monitoring/ evaluation framework (Mucha & Tharaney, 2013). Innovative resource mobilization to achieve and sustain financial resources commensurate to the need for intervention scale up is also enabling (Baker et al., 2018; Gillespie et al., 2013). In the short-term, local advocacy and targeted technical assistance can compensate for low strategic capacity at lower administrative (decentralized) levels. However, the effectiveness of such assistance depends on contextual ease of administrative processes, local funding and data, and existence of organizational and systemic capacity (Harris et al., 2016).

## Methods

### Study context

Changes in the nutrition enabling environment in Nigeria between 2008 and 2019 were assessed for the federal level and in two states. Representative state-level data for Nigeria became available in 2008; this time-point therefore allowed triangulation between empirical changes in nutrition determinants (Adeyemi et al., 2022) and perceived changes in the nutrition enabling environment.

Nigeria operates a federal structure; states are semi-autonomous and change across the country is driven by the efficiency and effectiveness of state-level processes (Akindele et al., 2002; Khemani, 2001). The thirty-six states and federal capital territory, Abuja, are grouped into six geopolitical zones (North Central, North East (NE), North West (NW), South East, South South, and South West) with an average of six states each. Human development in states within a geopolitical zone is relatively similar but development has consistently varied substantially in states across zones, with states in the three northern zones, especially the NE and NW, considerably lagging behind southern states (Archibong, 2018; Eze et al., 2014). The prevalence of nutrition determinants and outcomes also differs widely across states and zones (Adeyemi et al., 2022). To facilitate the understanding of nutrition enabling environments, we chose to contrast the environment in a state which had reported relatively little improvements in nutrition outcomes with a state that had better outcomes.

The NW zone has persistently had the highest prevalence of stunting in Nigeria. At the commencement of this study, in January 2019, Jigawa State in the NW had the highest prevalence of stunting in the country (66%, NBS & UNICEF, 2017) and stunting had increased from 53% in 2008 (NPC & Macro, 2009). We therefore selected Jigawa as the state with apparently little nutrition improvements. While states in southern zones had the lowest prevalence of stunting and the greatest stunting reductions over time, we chose to select a better performing state from the same geopolitical zone to minimize potential confounding due to the fundamental differences (Archibong, 2018; Eze et al., 2014) among states across zones. Kaduna was selected as the second state because it had the lowest stunting prevalence in the NW in 2017 and was the state in the zone that recorded the largest apparent improvement in stunting between 2008 and 2017 (stunting declined from 52 to 47%).

There are historical differences between Jigawa and Kaduna States. The current Kaduna State was formed in 1987 after some of its land area was carved out to form another state, but the area covered by Kaduna was already centrally governed in the precolonial era and Kaduna State’s capital was the capital of Northern Nigeria for many years in the colonial era (Wuam & Jatau, 2022). Jigawa State was formed out of another state in 1991. The two states differ in agroecological zones (Jigawa is more arid), religion/culture, economy, and other social, physical, and demographic characteristics (Adeyemi et al., 2022). Understanding factors associated with nutrition changes and challenges in these two states, considering the similar stunting prevalence in both states in 2008 as well as the divergent trajectories from 2008 to 2016, was therefore considered to be important for future efforts to improve nutrition in the zone and therefore Nigeria.

In addition to assessing past changes, the study captured enabling environment challenges that may hinder nutrition progress from 2019 to 2025.

### Enabling environment factors studied

The issues assessed by the study, guided by the components provided by Gillespie et al. (2013), are summarized in Table 2.

**Table 2:** Summary of Factors Assessed

**Enabling Environment Categories**	**Changes and Challenges Factors around Creating and Sustaining Momentum**	**Changes and Challenges Factors around Converting Momentum to Results**
Narratives, knowledge, and evidence	• Framing of ‘nutrition’ • Advocacy and focusing events • Evidence of interventions coverage, scale, and quality	• Research around what works contextually, and why and how • Impact pathways – clear expression of linkages between nutrition activities and attainment of goals
Political economy and governance	• Political attention • Multisectoral coordination, including role delineation and policy integration • Accountability mechanisms	• Vertical coordination of interventions delivery from federal to community levels • Civil society and private sector involvement in intervention delivery
Capacity and resources	• Leadership and championing • Systemic capacity – Existence of decision-making forums; timeliness, appropriateness, and effectiveness of information, financial, and decision flows • Strategic capacity – Soft power skills, including ability to envision a plan, build alliances, leverage resources, and mobilize commitment to achieving plan	• Delivery and operational capacity – Existence of adequate numbers of staff with appropriate knowledge, skill mix, and motivation; availability of money and tools necessary for service delivery; sufficient numbers of facilities (physical structures) • Resource mobilization

### Data sources

Data came from two sources: a policy review and interviews at the federal level and in Jigawa and Kaduna States.

#### Policy review

The policy review was undertaken to assess the documented policy landscape and changes in frameworks guiding nutrition interventions. Documents reviewed were identified and obtained through an electronic search as well as communication with key nutrition stakeholders in Nigeria. The electronic search used websites of the health, agriculture, environment, and water and sanitation ministries, departments, and agencies (MDAs), as these MDAs have mandates related to key actions for achieving optimal nutrition as conceptualized by (Black et al., 2013). A list of retrieved documents was shared with stakeholders to ensure that no relevant document was omitted and to retrieve any documents unavailable online. The review included policies, strategies, strategic frameworks, blueprints, or plans of action. Programme documents, guidelines, Acts, and Bills were excluded from the review because they derived from policies and strategies. Documents published from 1998 to 2019 were reviewed to ensure that all documents in use in 2008 were included (Nigeria’s policy cycle appeared to be about 10 years), as well as to cover multiple policy cycles and allow comparisons. To facilitate assessment of changes over time, documents were grouped into one of five time periods, based on the date of publication: 1996–2000, 2001–2005, 2006–2010, 2011–2015, and 2016–2019.

Forty-eight (48) documents were reviewed from the agriculture (4); economics (3); education (4); environment (5); health (17); water, sanitation, and hygiene (WASH, 4); nutrition (7); and other (4) sectors. Nutrition sector documents refer to documents that were published by any sector, but developed specifically for the goal of improving nutrition, and were typically initiated by the nutrition community. Online Resource 1 lists all reviewed documents by sector and year of publication.

#### Federal and state level interviews

Interview participants were selected from nutrition focal persons in various MDAs, staff of nutrition donor/development partner/United Nations agencies, personnel from Civil Society Organizations (CSOs), persons involved in nutrition monitoring and evaluation (M&E), and representatives from academia involved in national nutrition policy formulation and implementation. Participants were selected using snowball sampling, with initial participants being nutrition focal persons in MDAs. Recruitment and inclusion criteria for all participants comprised of being active in the national or state level nutrition sector since at least 2008 and able to talk about changes in nutrition from 2008 until 2019. Additional inclusion criteria were involvement in setting or influencing nutrition policy and programmes (federal level) and involvement in adapting national nutrition-relevant policies into state plans of action and managing programme implementation (state level).

Thirty-five (35) interviews were conducted – 16 at federal level, 10 in Jigawa, and 9 in Kaduna. Data was collected using separate, pre-tested, semi-structured interview guides for the federal and state level. All authors contributed to the development of the interview guides. Questions asked were informed by the enabling environment framework (Gillespie et al., 2013) and previous stories of change work in other countries (Gillespie et al., 2017). All interviews were conducted in English, by the first author, between May and December 2019, based on stakeholder availability. Table 3 summarizes the characteristics of the participants in the interviews. Four types of participants (government, NGO, development partner, and academia) were recruited from both the health and non-health sectors.

**Table 3:** Characteristics of Interview Participants

	**Federal**	**Jigawa**	**Kaduna**
***Total***	16	10	9
***Type of Participant***			
Government	6	7	5
NGO	2	3	3
Development Partners (Donors/UN)	4	–	1
Academia/Research	4 (2 North, 2 South)	–	–
***Sector of Experience***			
Health	5	6	5
Non-Health (agriculture, economic, education, environment, media) or multiple sectors	11	4	4

### Data analysis and validation of study findings

The federal and state-level interviews were audio-recorded and transcribed verbatim by two research assistants. Both policy documents and transcripts from the interviews were analysed in Nvivo 10, using a study codebook which was developed a priori and was pre-tested/revised using three each of policy documents and transcripts. Analysis was led by the first and last authors. All authors reviewed the themes and sub-themes that emerged from the data. Codes were based on the Gillespie et al. (2013) framework, and according to the factors summarized in Table 2. Analysis was conducted separately for the policy documents as well as for federal level and for each of the states. Themes were also reported separately for the documents and federal level and states. Themes that emerged across the assessed factors for federal, Jigawa, and Kaduna data were then compared and findings from documents and interviews were triangulated.

The study’s findings were validated in a 3-h virtual meeting facilitated by the last author, held December 2020, and attended by 20 out of the 35 participants interviewed. The objectives of the validation were to identify issues that may have been overlooked and inform final interpretations of study findings. The event was recorded and transcribed, and the transcript was subsequently coded and analysed in Nvivo 10 using the study codebook.

### Ethics

Ethics approval for the study was obtained from the National Health Research Ethics Committee (NHREC). Permission to conduct the study was additionally requested and received from the State Primary Health Care Development Agency in each state. Informed consent was obtained from all participants.

## Results

The results are summarized in Table 4 and are organized according to the categories in the framework used, and around factors highlighted in Table 2. For each category, changes from 2008 to 2019, as well as anticipated challenges to change from 2019 to 2025 are presented for factors to create and sustain momentum, as well as factors to convert momentum into results. Unless otherwise indicated, findings in Jigawa and Kaduna aligned with what was perceived at federal level, and results are reported together. The number of participants (n) that expressed viewpoints are noted in parentheses.

**Table 4:** Summary of Results

	**Narratives, Knowledge, and Evidence**	**Political Economy and Governance**	**Capacity and Resources**
**Changes around Creating and Sustaining Momentum**	Increased importance of nutrition in policies/strategies Nutrition accepted as multisectoral issue Advocacy increased and stimulated political attention to nutrition at all administrative levels Nutrition data representative at state level became available and increased Implementation of multisector interventions increased	Increased political attention for nutrition Increased number of nutrition actors and sectors of nutrition interventions More active multisectoral coordination platform and increased multisectoral dialogue at federal levels Increased multisectoral collaboration at state/LGA levels Increased accountability for nutrition change	Increased individual and organizational leadership and championing Inauguration of National Council on Nutrition Increased appointment of nutrition focal persons in non-health sectors
**Challenges around Creating and Sustaining Momentum**	Nutrition inadequately seen as a unique developmental challenge Advocacy approaches are inadequate Data quality limited, and some indicators have no data Intervention coverage low compared to magnitude of need	Political attention still inadequate and is unequal across states and LGAs Political attention may not be sustained from one political administration to another Limited sharing of information across sectors Duplication of coordination structures/roles Limited accountability mechanisms	Institutional arrangements potentially limiting systemic capacity Nutrition focal persons positioned at non-managerial levels in MDAs Limited technical and soft skills among nutrition focal persons
**Changes around Converting Momentum to Results**	Importance of implementation research now recognized	Increased adapting of national nutrition policies/strategies to address state peculiarities and priorities Increased state to LGA coordination in some states Increased civil society collaboration with public sector Increased private sector attention to nutrition	Increased nutrition budgeting and resource allocation at federal, state, and LGA levels Increased numbers of human resources for nutrition Increased training of frontline workers across multiple sectors
**Challenges around Converting Momentum to Results**	Conduct of implementation research is limited Impact pathways of policies/strategies are not explicit Little use of data to estimate potential effect sizes and justify interventions included in policies/strategies	Inadequate communication and engagement among federal, state and LGA levels Low involvement of state/LGA level actors in development of national policies/strategies Limited understanding of national policies at state/ LGA levels leads to limited adaptations and ownership Fragmented civil society actions Inadequate private sector engagement and accountability	Limited budgetary releases and cash backing Inadequate ability of nutrition actors to navigate funding bureaucratic processes Heavy reliance on donors for funding Funding focused on health sector and procurement of ready-to-use-therapeutic foods Inadequate numbers and knowledge/skills of human resources Inadequate work environments and logistics support Limited use of operational plans where they exist Stock out of commodities and tools High population growth strains capacity and limits improvements Physical insecurity limits service delivery and is increasing

### Framing, generation, and communication of knowledge and evidence

#### Creating and sustaining momentum

##### Framing of ‘nutrition’

**Fig. 1:**
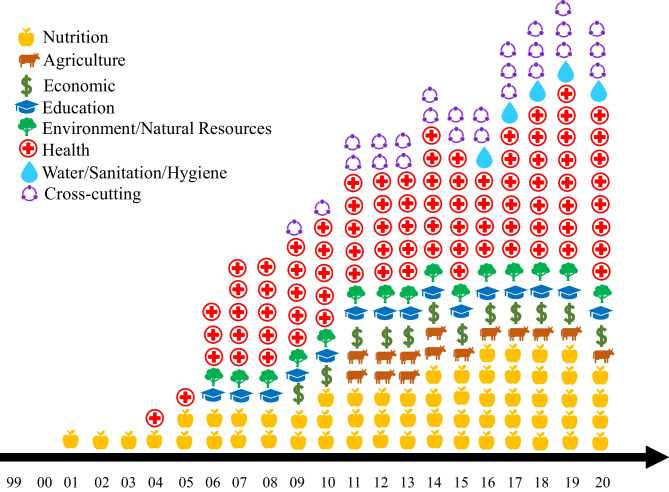
Number of Active Policies/Strategies in Nigeria that have Nutrition Objective(s), Activities, or Indicator(s), by Sector, from 1998 to 2019

The policy review found that the importance placed on nutrition rose over time as an increasing number of policies/strategies across all relevant sectors included nutrition objective(s), activities, and/or indicators (Fig. 1). Moreover, prior to 2010, nutrition sector documents were published just by the Ministry of Budget and National Planning. From 2010 however, nutrition documents were also published by Federal Ministry of Health (FMOH), and Federal Ministry of Agriculture and Rural Development. Online Resource 1 provides details about which documents include mention of nutrition, objective(s), activities, and/or indicators.

Still, some policies/strategies framed malnutrition solely as a cause or consequence of other developmental challenges, rather than as a development challenge in and of itself. The framing of malnutrition as a cause was reflected in documents that had nutrition objectives and/or activities but included no nutrition indicators in its monitoring and evaluation considerations. Examples of such documents include the 2006 *National School health Policy*, the 2017 *National HIV/AIDS Strategic Framework*, and the 2019 *National Health Promotion Policy*. The framing of nutrition as a consequence of other challenges was indicated by documents which emphasized nutrition as a problem to be addressed but did not include nutrition objectives or activities, suggesting a perception that nutrition will be addressed indirectly. Examples of such documents include the 2016 *Draft National Policy on the Environment* and 2017 *Economic Recovery and Growth Plan*.

Interview participants (*n =* 35) emphasized that there have been increases in the public and/or policymaker awareness about nutrition. Nutrition problem identification has changed; malnutrition used to be conceptualized as a problem arising from limited food availability, but a realization that states with the highest food production also have the highest undernutrition rates led many of the participants (*n =* 31) to highlight inadequate formal and/or nutrition/health education as a major determinant of inadequate dietary intakes and nutrition. However, some respondents (*n =* 20) expressed that food security was key to nutrition and that this was a challenge due to high and/or increasing poverty rates (*n =* 12). Other determinants mentioned by a few participants each included infection, low women’s empowerment, inadequate WASH, and poor food safety. Participants (*n =* 20) also noted that malnutrition used to be thought of as a health challenge, or primarily the responsibility of the health sector, but is now increasingly recognized to require multisectoral action. One health sector participant perceived that although other sectors were being mobilized to take a more active role in addressing nutrition; health was still the most important sector. Other participants mentioned that the understanding of dynamics among determinants of nutrition remained inadequate, and the bulk of efforts to address nutrition (by development partners or government) is still predominantly channelled through the health system and sector. Poverty reduction to facilitate financial access to nutritious foods was perceived to be key for future efforts to address undernutrition. A need for increased focus on behavioural attitudes and patterns that can lead to improvements in nutrition was also emphasized; due to the perception that nutrition change across multiple sectors (including WASH, use of income to purchase nutritious foods, etc.) requires behaviour change.

##### Advocacy and focusing events

Advocacy around nutrition was reported to have increased (*n =* 23), driving increased government awareness about nutrition, policy/strategy development, and budgetary allocations and releases, at federal, state, and local government area (LGA) levels. According to a participant “*High level advocacy is helping us to overcome nutrition challenges, because our leaders, they are ready to listen. Whenever there is advocacy on any issue, they listen and they take the appropriate action*” (JGS_009, government personnel). Federal level respondents (*n =* 9) mentioned that national and international events that have been key for encouraging greater attention to nutrition have also increased. Pivotal events mentioned included Nigeria’s signing up to the Scaling Up Nutrition (SUN) Movement in 2011; a National Nutrition Forum in 2012; the development of various national policies and strategies; the Malabo Declaration and revitalized Comprehensive African Agriculture Development Program (CAADP) framework; the release of the 2013 *Lancet Series on Nutrition*; the 2016 *Lancet Series* on Breastfeeding; World Health Assembly meetings; 2013 Nutrition for Growth Summit; 2014 International Conference on Nutrition, and declaration of UN Decade of Action on Nutrition. The heightened focus on nutrition in development fora, including fora about the Millennium Development Goals and the Sustainable Development Goals (SDGs), were also reported to be influential.

Despite the increased advocacy, gaps remain. A few federal level participants (*n =* 2) highlighted that the approach and intensity of advocacy efforts needed to improve. Nutrition actors did not always maximize opportunities to speak with political leaders and often emphasized the problem rather than required actions. Fact sheets were frequently used as an advocacy medium to political leaders, who often do not read the sheets, resulting in a situation whereby nutrition knowledge of political leaders is different from what the nutrition community thinks that they know.

##### Evidence of intervention coverage, scale, and quality

Participants reported that nutrition data that are representative at state levels increased since 2008, allowing for the assessment of trends in nutrition indicators at decentralized levels. Jigawa and Kaduna participants (*n =* 13) emphasized that the evidence provided by state level data had been instrumental in galvanizing and maintaining nutrition action. Participants explained that there has been increased commitment to implementation of nutrition programmes. For instance, Maternal, Newborn and Child Health Weeks (MNCHW), including vitamin A supplementation, were scaled-up and improved from 2008 to 2019. There was scale-up of community management of acute malnutrition (CMAM) intervention as well as promotion of and support for optimal infant and young child feeding (IYCF) from 2008 to 2019. In addition to health facilities, community structures are now being used to deliver health services such as antenatal care services. Several nutrition-sensitive social protection programmes were also introduced, including a national home-grown school feeding programme and a cash transfer programme. A few states, notably Lagos and Kaduna States, have ratified 6 months maternity leave to support exclusive breastfeeding. A micronutrient powder programme has been introduced. Biofortified staple crops and associated products have been introduced and their use is being expanded. Several investments are going into improved production of animal source foods, including fish.

Notwithstanding the perceived improvements in nutrition, study participants (*n =* 7) explained that intervention coverage was quite low compared to the magnitude of need. Some determinants of nutrition, including safe water, sanitation, waste disposal services and hygiene services were perceived to have achieved little to no progress. The low intervention coverage was highlighted in the study states. One Jigawa participant highlighted that, at the time of data collection, a locally developed multisectoral programme with high nutrition improvement potential (*Masaki* project) was covering just 150 out of the 12,000 communities in Jigawa. Study participants (*n =* 2) perceived that the affordability of nutritious foods has declined, and that the prevalence of stunting would have increased from 2013 to 2018 in the absence of already mentioned interventions (such as improved health services, management of acute malnutrition, and IYCF).

Although the generation of nutrition data increased from 2008, participants (*n =* 4, federal level) reported serious issues around data quality, including of routine data. Large differences in results of surveys designed to be representative of the same geographical areas have created uncertainties around what to believe about the nutrition situation. Also, some participants (*n =* 2, federal level) highlighted that available data is still inadequate, such that there are indicators (e.g., prevalence of various micronutrient deficiencies) for which no empirical information exists or information is extremely dated and does not allow assessment of time changes or impacts of interventions. State level participants (*n =* 2) remarked that there was inadequate representative data disaggregated at the LGA level.

#### Converting momentum to results

##### Contextual research

The policy review found that the importance of implementation research, including formative and/or operations research, was highlighted in more recent nutrition sector policies/strategies (2016 till 2019). Interview participants (*n =* 5) indicated that the conduct of research to inform implementation and decision making increased over the period studied. However, there is still inadequate research around how to implement already identified interventions, as well as identifying new and/or multisectoral interventions that can address the gaps in nutrition outcomes in Nigeria, vis-à-vis the context of different states and LGAs.

##### Impact pathways

The policy review found that the causal theory underlying nearly all the policy/strategy documents was not made explicit. In other words, it was not explained in the documents with nutrition objectives, activities, and indicators, how implementing policy roles and responsibilities would lead to achievement of objectives, or how achieving objectives would solve the expressed problem. The two exceptions were the 2014 *Health Sector National Strategic Plan of Action for Nutrition* (NSPAN), and the 2016 *Agricultural Sector Food Security and Nutrition Strategy* (AFSNS). Each of these documents provided an overarching theory of change that broadly explained the links between groups of activities and key objectives and links between the objectives and outcomes. In the 2014 NSPAN, UNICEF 1990 conceptual framework of malnutrition and Black et al. (2013) framework to achieve optimum foetal and child nutrition and development were stated to have guided the selection of activities in the Strategy. The 2016 AFSNS theory of change was targeted at explaining how the different actions in the AFSNS influenced key outcomes and pathways identified in an established causal framework (FIVIMS framework for the analysis of food security and nutrition). Additionally, there was limited use of data in selecting or justifying strategies and intervention areas, including in the 2016 *National Policy on Food and Nutrition*. Data was generally used to describe the prevalence and consequences of malnutrition, but not to estimate potential effect sizes of different interventions; except partially in the 2014 NSPAN where the aggregate potential impact of various packages of interventions were estimated. One interview participant emphasized the inadequate use of data to design and target interventions.

### Political economy of stakeholders, ideas, and interests

#### Creating and sustaining momentum

##### Political attention

Interview participants unanimously reported that there has been increased political attention for nutrition, evidenced by increased nutrition policies and programming, budgetary allocations, multisectoral (as opposed to health sector only) high level expression of commitment, and domestication of national policies at state level. The number of donors and organizations implementing nutrition actions, and the sectors in which interventions were being implemented, was also reported to have increased over time. Part of the increased attention to nutrition was attributed (*n =* 20) to nutrition champions at all levels of government. Participants (*n =* 3) mentioned that the guidance provided by national policies and federal level oversight had contributed to political attention for nutrition at state level. Nevertheless, participants (*n =* 15, mostly at federal level) considered that political attention for nutrition is still inadequate (evidenced by inadequate budgetary allocations and releases), is unequal across states, and may not be sustained from one political administration to another. Other participants spoke in positive terms about political attention gaining momentum and that advocacy should continue to keep the momentum. In the words of a participant, “*…so every year we see that government is becoming more committed; and even at the state level too, governments are now putting money into nutrition, so nutrition is budgeted for, and the funds are sometimes released*” (FLS_004, NGO).

##### Multisectoral coordination

In policies and strategies, roles were described at aggregate levels and did not specify which cadres of officers will perform which functions. Very few sectoral policies/strategies involved multisectoral stakeholders in their development and there was limited cross-referencing of complementary documents, suggesting limited coordination. Further, there appeared to be conflict around institutional roles for coordinating multisectoral nutrition actors. The Ministry of Budget and National Planning is the established national focal point for nutrition coordination and facilitates development and ratification of the *National Policy on Food and Nutrition* which is the overarching national framework for nutrition. Sectoral strategies, including the *Health Sector National Strategic Plan of Action for Nutrition*, are derived from this Policy. Yet, this Health Sector Plan of Action attributes the national coordination role to Federal Ministry of Health.

Findings from the interviews presented a mixed picture. Participants (*n =* 20) reported improvements in multisectoral dialogue and collaboration and highlighted several indications of improved multisectoral coordination. However, especially at the federal level, participants (*n =* 11) also emphasized that coordination is still weak. Indications of improvement included an expansion of the nutrition coordination team within Ministry of Budget and National Planning and ratification of a revised National Policy on Food and Nutrition in 2016. The National Committee on Food and Nutrition (NCFN), a platform for multisectoral nutrition-relevant MDAs to interact, improved and meetings were more frequently held (*n =* 7, federal). The Accelerating Nutrition Results in Nigeria project (a 350 million USD project facilitated by the World Bank) was initially planned to implement only health sector interventions. However, multisectoral consultations and advocacy led to the project incorporating interventions involving other sectors.

Coordination structures at state level, particularly the State Committee on Food and Nutrition (SCFN), have also been established/improved in many states (*n =* 7, federal), including in Jigawa and Kaduna States (*n =* 13). In both states, the SCFN coordinates multisector nutrition workplans and implementation. Both states also have additional coordination structures. Jigawa has a coordination Steering Committee for coordinating policies across sectors; with membership made up of Commissioners (Governor-appointed Heads) of relevant ministries (*n =* 6). In Kaduna, the Governor established the Kaduna Emergency Nutrition Action Plan (KADENAP) in 2016 and made his wife the Chairperson. A KADENAP Committee catalyses and fast-tracks implementation of workplans by SCFN members by facilitating cash backing of activities. The Kaduna State Budget and Planning Commission (SBPC) periodically convenes Development Partners’ coordination meetings, and meetings with health sector appointed LGA Nutrition Focal Persons. The Kaduna SBPC also facilitates assignment of intervention LGAs; so that rural (not just urban) areas receive services, coverage is increased, and there is minimal duplication of Development Partners within an LGA. Across the country and in both states, some LGAs now have active Local Government Committees on Food and Nutrition and improved LGA level multisectoral coordination (*n =* 13).

Challenges to effective multisectoral coordination included limited openness in sharing of workplans and implementation strategies across sectors, working in silos, and duplication of nutrition efforts. The *National Policy on Food and Nutrition* was criticised (*n =* 2) for not making the roles of sectors and institutions explicit. Some participants (*n =* 4, federal) did not think the NCFN improved within the study period because they perceived that a lot of talk happened at the meetings but little action or actual coordination thereafter. A few participants (*n =* 5, federal level but 1) also perceived that though nutrition coordination has been statutorily assigned to the Ministry of Budget and Planning, the health sector wants the overall leadership and coordinating role; even though nutrition coordination within the health sector itself was reported to be inadequate (*n =* 4, federal level). One participant in the health sector explicitly perceived the ministry of health to be the best home for nutrition coordination because Federal Ministry of Health (FMOH) is the Secretariat for the Scaling Up Nutrition (SUN) Movement coordination; and the health sector was perceived to have more nutrition interventions, human resources, technical knowhow, funding, and other capacity than other sectors. The Minister of Health was the government representative to sign Nigeria to SUN Movement, so the Head of Nutrition at FMOH was appointed as SUN focal person by default.

Overall, federal participants (*n =* 3) perceived that there was inadequate understanding of the SUN Movement coordination system and effectiveness, including vis-à-vis coordination by Ministry of Budget and National Planning, and that nutrition coordination structures were duplicated. Other participants (*n =* 5) considered that the FMOH is not the right home for a SUN focal person and that the position should be moved to the Presidency or the Ministry of Budget and National Planning. A coordination challenge peculiar to Jigawa was the inability of the Steering Committee to meet regularly because of the very busy schedule of its members, leading to reduced effectiveness of the Committee (*n =* 1). Challenges to multisectoral coordination were described by a participant in this way “*…[nutrition] is prone to conflict. It is prone to derailing of objectives. It is prone to…there is one other word… I’ve forgotten it, but it has to do with starting at a point and then at the end of the day, you find out that you ended up somewhere else because stronger pulls of some very strong groups within that system insisted that things must go their way*” (FLS_012, academia).

##### Accountability mechanisms

Increased involvement of legislators in nutrition accountability of government and development partners was reported by participants (*n =* 9) at federal level and in both states. In Jigawa State, it was mentioned (*n =* 4) that the media and CSOs had also been engaged for budget tracking and accountability for nutrition actions. In Kaduna, KADENAP holds MDAs accountable for nutrition (*n =* 3). Further, both states highlighted (*n =* 9) ways that had been used to increase accountability for nutrition at the LGA level, including mandating specified budgets for nutrition and withholding these monies at state level before disbursing LGA allocations.

A few federal participants (*n =* 2) however highlighted that accountability mechanisms at all levels of government were generally inadequate, including for the allocation and management of financial resources. Compliance with appropriate actions for nutrition was not rewarded and there were also no sanctions for inaction at any level.

#### Converting momentum to results

##### Vertical coordination

Policies/strategies described that mechanisms for vertical coordination include National Councils that exist in every sector. National Councils convene annually and bring together the Honourable Minister, Commissioners from each of the 36 states in Nigeria and the Federal Capital Territory, other high-level state representatives, representatives of key development partners, and experts, for a sector. National Councils initiate and/or recommend policy/strategy formulation in a sector, approve and adopt formulated policies, provide a forum for coordination, facilitate discussion of critical sectoral issues and mediation of issues, ensure compliance with agreements and commitments, and review performance.

Study participants (*n =* 15) reported that aspects of vertical coordination of nutrition have improved, with more states now domesticating national nutrition policies by adapting them to state priorities, context, and peculiarities. State participants (*n =* 7) highlighted that national policies developed by the federal level provided guidance to states for adapting policies/strategies. State participants (*n =* 13) likewise perceived positive changes had occurred for state to LGA level coordination. For instance, Kaduna participants (*n =* 2) reported greater vertical coordination in the health sector. Health workers in primary health care centres, since 2017/2018, have their salaries shared between the LGA and state governments, increasing their accountability to the State Primary Health Care Development Agency; unlike in the past when they were accountable only to their LGA (primary health care workers are statutorily hired at the LGA level). Two participants further highlighted some existing LGA to health facility and community level coordination processes. In Jigawa, health sector coordination has been improved by having Nutrition Focal Persons in every LGA report to the State Nutrition Officer and a Deputy State Nutrition Officer.

Still, vertical coordination was largely referred to by federal participants as inadequate or requiring additional efforts/new approaches (*n =* 6) or difficult/ineffective (*n =* 4), as well as non-existent (*n =* 2). There is limited involvement of state and LGA actors in federal level policy/strategy development and the constitutional nutrition autonomy of states and LGAs means that there is generally no obligation for state MDAs to report to federal MDAs. Inadequate communication and engagement (in both directions) between one level and the next fuels limited understanding and domestication, and thus insufficient ownership and implementation of policies. There are further limited incentives for improved implementation. In the words of a participant, “*unfortunately, [vertical coordination] does not exist. The states are independent. The federal is independent. The local government is independent……… the president cannot tell any governor – this is what I want you to do. He can only advise; he can only suggest. …It’s only moral suasion that can be used to convince.*” (FLS_002, development partner). One participant perceived that “*local government level coordination is almost non-existent*”, but that there is no need for coordination at that level, since they work readily with the state level. Another participant perceived that challenges with vertical coordination were partly due to a lack of clarification of nutrition roles for each level of government.

##### Civil society and private sector involvement

At federal level and in both states, participants (*n =* 28) mentioned that CSOs have supported public-sector capacity development to facilitate nutrition awareness creation, advocacy, development of operational plans, resource mobilization, budget tracking and accountability mechanisms, or implementation by frontline workers. Participants (*n =* 7) mentioned that the Civil Society Network for Scaling Up Nutrition in Nigeria (CS-SUNN) had emerged over the study period and had played a key role in bringing CSOs together, particularly around common advocacy, resource mobilization, and budget/ implementation tracking tools. Still, one participant noted that CSOs actions had not achieved as much success as they could have because the efforts were fragmented; while another participant criticized that CS-SUNN is not structured to coordinate CSOs but rather acts like a CSO itself.

Attention and contribution of the private sector to nutrition likewise improved, especially as evidenced by increasing commitment to food fortification (*n =* 5), and the establishment and functioning of the SUN Business Network (*n =* 4). Nevertheless, participants (*n =* 6) remarked that there is a need to intensify focus on the potential of the private sector to improve nutrition, so that, for example, there is greater compliance with fortification standards and six months paid maternity leave and/or child-friendly workplaces are supported.

### Capacity (individual, organisational, systemic) and financial resources

#### Creating and sustaining momentum

##### Leadership and championing

Participants highlighted that there are now many nutrition champions, unlike previously when UNICEF was the only apparent champion. The most frequently mentioned individual champions were the Chairman of the Legislators' (federal or state) Committee on Health (*n =* 7), Emir of Kano 2014–2020 (*n =* 5), and the Minister of Agriculture 2011–2015 (*n =* 5). Organizational champions commonly mentioned included UNICEF (*n =* 19), Save the Children International (*n =* 10,), UK Department for International Development (*n =* 9), and Bill and Melinda Gates Foundation (*n =* 8). Other champions mentioned (*n =* 11) were the wives of political leaders who had adopted nutrition activities as a pet project, such as the wife of Kaduna State Governor. Following advocacy efforts in 2015, the Kaduna State Governor frequently made public pronouncements about nutrition and acknowledged the state’s nutrition problem, and his wife became instrumental in galvanizing nutrition change in the state (*n =* 9).

##### Systemic capacity

Participants emphasized that the 2017 inauguration of the National Council on Nutrition (NCN) was a major milestone (*n =* 11, federal). NCN had been established in 2007 to be the highest decision-making, coherence, and accountability body for nutrition, but was not inaugurated and was thus non-functional. The NCN is chaired by the Vice President of Nigeria and meetings convene the Ministers of all nutrition-relevant ministries. Capacity improvements can be summed up in this quote: “*……there are powerful structures and mechanisms and funding opportunities that are created that were not there before, which shows that this is a huge moment to really advance nutrition issues*” (FLS_006, development partner).

Notwithstanding, a few participants (*n =* 2) considered that the potential effectiveness of the NCN may have been reduced due to the creation of Ministry of Budget and National Planning. When the NCN was established in 2007, what is now the Ministry of Budget and National Planning was the National Planning Commission and was appointed to be the NCN Secretariat. The Vice President of Nigeria Chaired the Commission as well as the NCN, so there was no problem with the NCN Secretariat being in the Commission. The Commission was however converted to a Ministry in 2015 and the Vice President cannot chair a Ministry. Hence, it may be necessary for the NCN Secretariat to be moved to the Office of the Vice President if an effective NCN is to be achieved.

##### Strategic capacity

Participants reported improvements in strategic capacity across government institutions. At the federal level (*n =* 12), the Federal Ministry of Agriculture and Rural Development has had leadership to drive a nutrition agenda and funding and saw changes in nutrition capacity such as a 10-year nutrition strategy, and establishment of a nutrition division and assignment of staff. Other nutrition-relevant ministries (for instance ministries of information, women affairs, and water resources) that did not experience as many significant nutrition changes now have a nutrition desk officer (*n =* 3). This increased appointment of nutrition focal persons in non-health sectors also occurred at state level (*n =* 6) and in some LGAs (*n =* 1). More LGAs now have a health sector nutrition focal person (*n =* 6).

Nonetheless, there is limited capability for nutrition focal persons within MDAs to think critically and strategize about how best to address nutrition (*n =* 13). Relatedly, nutrition responsibilities need to be assigned to higher ranking officers within key institutions at federal and state levels; officers that have convening power to mobilize and coordinate nutrition actions within their MDA (*n =* 5).

#### Converting momentum to results

##### Resource mobilization

Federal government budgeting and funding for nutrition increased since 2008, with improved attention to budgetary releases (*n =* 11); donor funding similarly increased (*n =* 8). More nutrition-relevant sectors and MDAs have started to budget for nutrition activities at both federal and state level and there have been new nutrition programmes (*n =* 12). In Jigawa, participants (*n =* 3) stated that nutrition budget lines have been created in several MDAs, including agriculture, education, health, budget and planning, and women affairs. From 2008 to 2017, the State did not release any funds for nutrition, except for nutrition services delivered as part of free maternal, newborn and child health services. However, from 2017, release of funds for procurement of ready-to-use therapeutic foods commenced and an increased number of CMAM centres were established. LGA Chairmen have also commenced fund release for CMAM and infant and young child nutrition (IYCN) counselling (*n =* 4). Intensive efforts to scale-up nutrition actions in Jigawa commenced in 2018, including the introduction of *Masaki* (*n =* 6). In Kaduna, workplans for nutrition are funded at both state and LGA levels (*n =* 4). Since at least 2001, Kaduna had a non-specific budget line in the State Budget and Planning Commission, through which any SCFN member could obtain funding for nutrition activities (*n =* 6). Since 2014 and 2018, there has been at least one budget line specifically for nutrition at state and LGA level, respectively (*n =* 3). There have also been increases in release of budgeted funds and cash-backing of released funds (*n =* 3). Further, resource allocation has recently prioritized preventive (IYCF counselling) over curative (CMAM) interventions (*n =* 1).

Nonetheless, the government’s political attention to nutrition is yet to be matched with commensurate financial investments (*n =* 8). Inclusion of nutrition in government budgets was criticised as often being rhetorical because of limited release/cash backing of funds (*n =* 14). Government funding for nutrition also appeared more discretionary than funding for other matters (such as vaccination) and was frequently re-appropriated when there was need for extra-budgetary funds (*n =* 2). Besides, at state and LGA levels, especially in non-health sectors, available funds were sometimes not accessed because of officers’ inability to develop clear activities, put together a memo to request for funds, complete approval processes, secure funds, and implement activities (*n =* 4). Funding is also still heavily donor dependent (*n =* 8) and development partners tended to focus on funding nutrition within the health sector, while nutrition activities in other sectors receive much less attention (*n =* 2). Within the health sector, the bulk of resources goes to procurement of ready-to-use-therapeutic foods for treatment of severe acute malnutrition (*n =* 12) and preventive services are more underfunded (*n =* 4). Apart from obtaining funds, judicious use of funds was a challenge (*n =* 2).

##### Delivery and operational capacity

A few participants mentioned that nutrition workforce capacity increased at federal level and in both states, with increased recruitment of human resources and capacity development of formal and informal frontline workers across multiple sectors (*n =* 3). For instance, in Jigawa, one agriculture extension worker is involved in implementing nutrition-sensitive agricultural activities per LGA (*n =* 1).

Mostly, participants reported challenges with delivery and operational capacity. Participants (*n =* 15) perceived inadequate human resources (numbers and quality), in addition to funding, to be the limiting factor for scale-up of interventions that will improve nutrition indices. Generally, implementation quality reduces as one moves from federal to community level, due to dwindling physical and other infrastructure and numbers and quality (technical knowledge and/or skill) of human resources (*n =* 1). There is further poor management of available human resources, with unconducive work environments, inadequate logistics support, poor remuneration, and limited opportunities for continuing education (*n =* 8). There was inadequate use of available community platforms to synergize activities and reduce costs (*n =* 3). Government personnel often developed operational plans based on the influence of development partners and such plans were frequently not used (*n =* 1). The health sector, perceived as the major nutrition actor at state level, was reported to be very weak, delivering poor quality of service and inadequately able to support nutrition programmes (*n =* 5). Other operational challenges included stock outs of commodities, inadequate supervisory capacity, and poor information management systems (*n =* 3). High population growth has additionally been a challenge for increasing and improving service delivery (*n =* 3, states). Physical security was further highlighted as an extremely critical factor affecting the implementation and scale-up of nutrition interventions and currently increasing insecurity is likely to increase undernutrition (*n =* 6).

Participants at the validation meeting stated that the SoC findings resonated with ongoing discussions at National Committee on Food and Nutrition meetings chaired by the Vice President of Nigeria. The study team was urged to send a one-page summary of findings and recommendations to the Office of the Vice President, ahead of pending Committee meetings. The subsequently prepared and shared document emphasized role definition and joint operational planning processes that will enable simultaneous delivery of multisectoral interventions to populations at risk of malnutrition. The National Committee on Food and Nutrition has since taken steps to get relevant sectors to agree to implement a common results framework and joint work plan based on a newly launched National Multisectoral Plan of Action for Food and Nutrition. The Vice President has established a Technical Working Group to define nutrition roles and responsibilities for sectoral ministries; develop annual work plans, advocacy, and communication materials; and facilitate capacity development for the different ministries to perform their roles.

## Discussion

This study aimed to describe the changes in the Nigeria nutrition enabling environment between 2008 and 2019 and identify areas of improvement for scaling up nutrition interventions. Overall, at both state and federal levels, the study found that political attention for nutrition increased, and there was a significant increase in the number and scope of nutrition-relevant policies/strategies and actors. Other enabling environment factors that improved included increased acceptance of nutrition as a multisectoral issue, data, advocacy, accountability, coordination structures, leadership, funding, and implementation of nutrition interventions. Critical challenges remain around operationalizing multisectoral and vertical coordination mechanisms, increasing quantity and quality of capacity (human, operational, and financial), and rapid scale-up of multisector interventions.

The importance of nutrition enabling environments are amplified in our study through the disparities in the environment in Jigawa and Kaduna States. As highlighted in the introduction and methods, other authors already documented poorer nutrition outcomes and determinants in Jigawa (Adeyemi et al., 2022). Our study found key differences in leadership, funding, and multisectoral and vertical coordination between the two states. Even prior to the period covered by our study, all relevant sectors in Kaduna had access to some funds for nutrition. Although both states reported increased political commitment for nutrition in recent times, changes occurred earlier in Kaduna and the level at which commitment occurred, and thus the leadership for driving change, diverged. In Kaduna, the State Governor, who is the highest constituted authority for the state, and his spouse, were strongly committed to nutrition. This leadership resulted in nutrition being generally prioritized. In Jigawa, such high-level commitment was not reported, although commitment increased among the civil service leadership and legislators. Further, the multisectoral coordination mechanisms in Kaduna had been active longer than that in Jigawa. Kaduna had also changed mechanisms of vertical coordination in the health sector to increase efficiency, effectiveness, and accountability. It is also possible that non-studied and historical differences between the two states, as highlighted in the methods section, played a role. However, since these differences existed before 2008 and had not prevented stunting rates from being similar in 2008, 53% and 52% in Jigawa and Kaduna, respectively (NPC & Macro, 2009), the role of better enabling environments cannot be dismissed. Still, the intervention coverage in Kaduna appears to have stagnated in recent years and stunting in the state is still well above the national average (Adeyemi et al., 2022). Moreover, Kaduna has a much higher population and possibly higher population growth rate than Jigawa (NBS, 2018), indicating a greater burden for current and future service delivery. Hence, it is important to improve the enabling environment and intervention delivery in both states.

Unlike previous nutrition stories of change studies, we studied enabling environment in a country that had not experienced considerable progress in stunting reduction. Yet, our findings correspond with the conclusions of the earlier studies (Bhutta et al., 2020; Gillespie et al., 2017; Heidkamp et al., 2021) and highlight a few key issues. Our findings show that nutrition in Nigeria has come a long way from the period reported by other authors (Benson, 2007, 2008) when nutrition was not at all prioritized. Nevertheless, we also show that increase in political attention, even when backed by increased policies and strategies, is not sufficient to create and sustain nutrition change or convert change to results. These findings concur with recent work on political commitment (Baker et al., 2018, 2019; Fracassi et al., 2020). Increase in expressed political attention and policies/strategies reflect rhetorical and institutional commitment, respectively, and are insufficient to generate sustained commitment to addressing nutrition and achieving target outcomes (Baker et al., 2018, 2019; Fracassi et al., 2020). Operational, embedded, and system-wide commitment, which encompass adequate financial, human, and technical resources as well as effective coordination at all levels, among other factors, are also indispensable. Yet, these commitment factors experienced more challenges than improvements during the period covered by our study.

Our study highlights the importance of understanding policy environments as a prerequisite for achieving progress in nutrition goals. Without understanding the conditions needed to create enabling environments conducive to nutritional improvements, nutrition actions may tend towards identifying and initiating new policies/programmes without addressing fundamental issues that limit implementation and effectiveness of such policies/programmes. To improve the nutrition enabling environment in Nigeria, vis-à-vis the characteristics of effective such environments, we summarise the following actions that stakeholders felt to be most pertinent during validation of our analysis.

Firstly, to make narratives, knowledge, and evidence more enabling, greater attention needs to be paid to unifying narratives and framing of nutrition issues and generating credible data that will facilitate a shared understanding of the issue and its severity. The differing perspectives of participants in our study about the contributory factors and solutions to malnutrition indicate that evidence about the contributions of various determinants to malnutrition in Nigeria is either unavailable or is not widely disseminated. Policies and wider government strategies will also need to incorporate such data in the selection of focus interventions and in determining achievable targets. The need for ‘SMART’ objectives^3^, is all the more important given the disconnect between a good level of high-level political rhetoric on action and the thin progress made in several key areas of implementation documented here and in the outcomes and determinants charted in Table 1.

Secondly, to improve political and governance structures and to achieve wider systemic capacity (and as emphasised repeatedly in our interviews), Nigerian nutrition actors need to explicitly define the contextual goals and mechanisms for multisectoral and vertical coordination and communicate these goals and mechanisms to actors at all administrative levels. The importance of this action is underscored by our finding that some current coordination practices may be counterproductive for nutrition. For instance, the coordination of donors in Kaduna State focuses on ensuring a spread of multisector donors across LGAs, rather than a concentration of donors in specific locations. This coordination approach suggests that support for complementary nutrition determinants is systematically not delivered to communities and households concurrently (i.e., there is no co-location), contrary to recommendations for nutrition progress (Heidkamp et al., 2021; Levinson et al., 2013).

Finally, to improve the necessary individual, organizational, and systemic capacity and human resources for implementing interventions with fidelity and at scale, our findings indicate the need for a national nutrition workforce strategy and implementation plan to guide improvement of nutrition training programmes and academic curricula, and then the rollout of the improved training to in-service and pre-service nutrition-relevant human resources. The workforce strategy must also include necessary, complementary, institutional and organizational capacity development (Gillespie et al., 2013; Potter & Brough, 2004), with discussion and agreement from key stakeholders on achievable nutrition roles for various institutions and personnel, across all administrative levels, taking into account existing structures and responsibilities as part of a genuine assessment of capacity. Additional key steps for making capacity and resources enabling for nutrition include the sequencing and prioritization of interventions needed across relevant sectors.

Our study highlights some research gaps. Several accounts indicate that a primary starting point for initiating actions to change nutrition enabling environments is the understanding and strengthening of actor networks (Baker et al., 2018, 2019; Namugumya et al., 2020a; Pelletier et al., 2018). While our paper provides information about Nigeria nutrition actor networks, it does not directly describe or characterize the networks. Also, our policy review highlights aspects of the nutrition policy integration in Nigeria but does not cover all dimensions of policy integration (Namugumya et al., 2020b). Specifically, our study did not assess the scope of policy goals, i.e., whether all nutrition determinants are being addressed across ratified policies/strategies. We also did not address whether strategies being implemented align with the nutrition problems in the country.

## Conclusion

Nutrition enabling environments are a critical limiting factor for generating political commitment for nutrition in all its forms and ensuring/sustaining high coverage of multisectoral interventions needed to improve nutrition. Our study describes changes in the nutrition enabling environment in Nigeria from 2008 to 2019 and identifies improvement needed in the enabling environment to accelerate scale-up of nutrition interventions and progress in achieving nutrition targets. Overall, several aspects of the enabling environment improved between 2008 and 2019 and facilitated increased implementation of nutrition-specific and nutrition-sensitive interventions, including CMAM, optimal IYCF promotion, vitamin A supplementation, and nutrition-sensitive health services. Enabling environment components that improved included the framing of nutrition as a multisectoral issue, nutrition advocacy, political attention, evidence around intervention coverage, civil society involvement, and activity of nutrition champions. The findings highlight that Nigeria has come a long way from having little political commitment for nutrition in the early 2000’s and that rhetorical and institutional commitment to nutrition has considerably increased. These factors have been especially important in creating and sustaining momentum for addressing malnutrition.

Although challenges still exist in these aspects, more challenges persist for factors needed to convert momentum into nutrition outcome results and achieve operational, embedded, and system-wide commitment for nutrition in Nigeria. Especially, limited operationalization of existing mechanisms for multisectoral and vertical coordination, inadequate technical and other skills among nutrition policy and program planners, inadequate numbers of frontline workers, and financial resources that are not commensurate with the magnitude of need, constrain enabling environments for nutrition in Nigeria. Addressing these challenges in a timely manner will be especially important for achieving future progress in nutrition in Nigeria.

## Supplementary Information

Below is the link to the electronic supplementary material.

Supplementary file1 (DOCX 31 KB)

## Footnotes

1 Transform Nutrition West Africa is a regional platform focused on enabling effective policy and programmatic action on nutrition (https://westafrica.transformnutrition.org/).

2 Other Stories of Change studies carried out under TNWA were in Burkina Faso and Ghana (Aryeetey et al., 2022; Becquey et al., 2022).

3 Specific, Measurable, Achievable, Relevant, Time bound.
