# Supplementary material for: Changes in Nigeria’s enabling environment for nutrition from 2008 to 2019 and challenges for reducing malnutrition
Source: Food Secur. 2022 Nov 22;15(2):343–61. doi: 10.1007/s12571-022-01328-2 (PMC9684792; doi:10.1007/s12571-022-01328-2)
Supplement: Supplementary file 1 — Supplementary file1 (DOCX 31 KB) [file 12571_2022_1328_MOESM1_ESM.docx]

**Journal: *Food Security***

**Changes in Nigeria’s Enabling Environment for Nutrition from 2008 to 2019 and Challenges for Reducing Malnutrition**

Olutayo Adeyemi^1,2^, Mara van den Bold^3,4^, Nicholas Nisbett^5^, Namukolo Covic^3,6^

^1^Department of Human Nutrition and Dietetics, Faculty of Public Health, University of Ibadan, Ibadan, Nigeria

^3^Formerly of the International Food Policy Research Institute, Washington D.C.

^4^Clark University, Worcester, Massachusetts, USA

^5^Institute of Development Studies, Brighton, U.K.

^6^International Livestock Research Institute, Addis Ababa, Ethiopia

^2^Corresponding Author: Email – [adeyemiolutayo@gmail.com](mailto:adeyemiolutayo@gmail.com); ORCID – 0000-0002-5066-517X

Running Page Title: Nigeria Nutrition Enabling Environment

# Online Resource 1: Policies and Strategies Reviewed by Sector and Year of Publication

|  | **Sector** | **Name** | **Year** | **Mentions Nutrition** | **Nutrition Objective(s)** | **Nutrition Activities** | **Nutrition Indicator(s)** |
| --- | --- | --- | --- | --- | --- | --- | --- |
|  | Agriculture | New Agricultural Policy Thrust | 2001 | No | No | No | No |
|  |  | National Agricultural Investment Plan 2011 – 2014 | 2010 | Yes | Yes | Yes | Yes |
|  |  | Agricultural Transformation Blueprint (2011 – 2015) | 2011 | Yes | Yes | Yes | No |
|  |  | Agriculture Promotion Policy 2016 – 2020 | 2016 | Yes | Yes | Yes | No |
|  | Economic | National Economic Empowerment and Development Strategy (NEEDS, 2003 – 2007) | 2004 | Yes | No | No | No |
|  |  | Nigeria Vision 20:2020: Economic Transformation Blueprint (2009 – 2020) | 2009 | Yes | No | No | Yes |
|  |  | Economic Recovery and Growth Plan 2017 – 2020 | 2017 | Yes | No | No | No |
|  | Education | National Policy on Education 4th Edition | 2004 | Yes | No | No | No |
|  |  | National Policy on Gender in Basic Education (2006 – 2015) | 2006 | Yes | No | No | No |
|  |  | National School Health Policy | 2006 | Yes | Yes | Yes | No |
|  |  | National Policy on Education 6th Edition | 2013 | Yes | No | No | No |
|  | Environment | Draft National Policy on the Environment | 1999 | Yes | No | No | No |
|  |  | National Forest Policy | 2006 | Yes | Yes | No | No |
|  |  | Great Green Wall for the Sahara and Sahel Initiative: National Strategic Action Plan (2012 – 2017) | 2012 | Yes | No | No | No |
|  |  | National Biodiversity Strategy and Action Plan 2016 – 2020 | 2015 | Yes | No | No | No |
|  |  | Draft National Policy on the Environment (Revised 2016) | 2016 | Yes | No | No | No |
|  | Health | Revised National Health Policy | 2004 | Yes | No | No | Yes |
|  |  | National Health Promotion Policy | 2006 | Yes | No | Yes | Yes |
|  |  | National Child Health Policy | 2006 | Yes | Yes | Yes | Yes |
|  |  | Integrated Maternal, Newborn and Child Health Strategy (2007 – 2015) | 2007 | Yes | No | Yes | Yes |
|  |  | National Policy on the Health and Development of Adolescents and Young People in Nigeria (2007 – 2015) | 2007 | Yes | No | No | Yes |
|  |  | National Strategic Health Development Plan Framework 2009 – 2015 | 2009 | Yes | No | No | No |
|  |  | National Policy on Food Safety | 2014 | Yes | No | No | No |
|  |  | National Health Information System Strategic Plan 2014 – 2018 | 2014 | Yes | No | No | No |
|  |  | Task-Shifting and Task-Sharing Policy for Essential Health Care Services in Nigeria | 2014 | Yes | No | Yes | No |
|  |  | National Health ICT Strategic Framework 2015 – 2020 | 2016 | Yes | No | No | No |
|  |  | National Health Policy 2016 | 2016 | Yes | Yes | Yes | Yes |
|  |  | National Reproductive Health Policy 2017 | 2017 | Yes | No | Yes | Yes |
|  |  | National HIV/AIDS Strategic Framework 2017 – 2021 | 2017 | Yes | No | Yes | No |
|  |  | National Action Plan for Health Security 2018 – 2022 | 2018 | No | No | No | No |
|  |  | Second National Strategic Health Development Plan 2018 – 2022 | 2018 | Yes | Yes | Yes | Yes |
|  |  | National Health Promotion Policy | 2019 | Yes | No | Yes | No |
|  |  | National Multi-sectoral Action Plan for the Prevention and Control of Non-Communicable Diseases 2019 – 2025 | 2019 | Yes | No | Yes | Yes |
|  | Nutrition | National Policy on Food and Nutrition in Nigeria (2001 – 2016) | 2001 | Yes | Yes | Yes | Yes |
|  |  | National Plan of Action on Food and Nutrition in Nigeria (2005 – 2015) | 2004 | Yes | Yes | Yes | Yes |
|  |  | National Policy on Infant and Young Child Feeding in Nigeria | 2010 | Yes | Yes | Yes | No |
|  |  | National Strategic Plan of Action for Nutrition: Health Sector Component 2014 – 2019 | 2014 | Yes | Yes | Yes | Yes |
|  |  | Agricultural Sector Food Security and Nutrition Strategy 2016 – 2025 | 2016 | Yes | Yes | Yes | Yes |
|  |  | National Social and Behavioural Change Communication Strategy for Infant and Young Child Feeding (IYCF) in Nigeria 2016 – 2020 | 2016 | Yes | Yes | Yes | Yes |
|  |  | National Policy on Food and Nutrition in Nigeria (2016 – 2025) | 2016 | Yes | Yes | Yes | Yes |
|  | WASH | National Water Policy | 2004 | No | No | No | No |
|  |  | Draft National Water Sanitation Policy (2004 – 2025) | 2004 | No | No | No | No |
|  |  | Draft National Water Policy (2016 – 2030) | 2016 | Yes | No | No | No |
|  |  | Partnership for Expanded Water Supply, Sanitation and Hygiene (PEWASH) Strategy (2016 – 2030) | 2016 | Yes | Yes | No | Yes |
|  | Other | National Gender Policy Strategic Framework 2008 – 2013 | 2008 | No | No | No | No |
|  |  | Second National Youth Policy | 2009 | Yes | No | Yes | No |
|  |  | Science, Technology and Innovation (STI) Policy | 2011 | Yes | No | Yes | No |
|  |  | National Social Protection Policy | 2017 | Yes | Yes | Yes | No |
